# Effect of Biodegradable PLA-Based and Conventional LDPE Mulch Films on Pathogenic and Functional Soil Microbial Communities

**DOI:** 10.3390/ijms27125530

**Published:** 2026-06-18

**Authors:** Magdalena Zaborowska, Jadwiga Wyszkowska, Agata Borowik, Jan Kucharski

**Affiliations:** Department of Soil Science and Microbiology, Faculty of Agriculture and Forestry, University of Warmia and Mazury in Olsztyn, 10-719 Olsztyn, Poland; m.zaborowska@uwm.edu.pl (M.Z.); agata.borowik@uwm.edu.pl (A.B.); jan.kucharski@uwm.edu.pl (J.K.)

**Keywords:** microplastics, PLA mulch film, LDPE mulch film, soil bacteriobiome, pathogenic bacteria, humic acids, bioaugmentation

## Abstract

Plastics and microplastics are widespread in the environment, yet knowledge about their impact on agricultural soils, including their microbiological properties, remains limited. Therefore, this study addressed the research question regarding the impact of secondary microplastics, biodegradable poly(lactic acid) (PLA) mulch film, and low-density polyethylene (LDPE) film on the abundance, structure, and functions of soil bacteria, with particular emphasis on the presence of bacterial pathogens. PLA and LDPE were applied to the soil at a dose of 4 g kg^−1^ d.m. of soil. The aim of the experiment was to evaluate and compare the effectiveness of soil bioaugmentation with the *Pseudomonas umsongensis* strain and its biostimulation with humic acids in mitigating the negative effects of microplastics. The response of culturable bacteria revealed high sensitivity of organotrophic bacteria to both microplastics, with a stronger inhibitory effect from PLA, as well as stimulation of actinomycetes. 16S rRNA gene amplicon sequencing indicated that the materials differentially influenced the bacterial response. PLA most strongly stimulated *Actinobacteriota* and favored the dominance of *Bacillus* and *Limnochorda*, whereas LDPE promoted the growth of *Actinobacteriota* and *Chloroflexota* as well as genera *KD4-96* and *1921-2*. Both microplastics were colonized by potential pathogens, including *Bacillus*, *Mycobacterium*, *Ralstonia*, and *Cupriavidus*. PLA additionally stimulated the proliferation of *Leifsonia* sp. and *Curtobacterium* sp., while both PLA and LDPE reduced the abundance of *Enterobacter* sp. and *Herbaspirillum* sp. Bioaugmentation using the *Pseudomonas umsongensis* strain was more effective in restoring the balance of the soil microbiome than biostimulation with humic acids. The results indicate that microbial preparations based on *Pseudomonas umsongensis* may serve as an important tool in restoring the balance of soil exposed to microplastics.

## 1. Introduction

It is undeniable that plastics are widely used in modern society. Due to their durability, affordability, and versatility, global production has reached an alarming level of over 480 × 10^9^ kg per year [1], while the recycling rate stands at a mere 9% [2]. It is estimated that by 2050, modern technologies for the production and recovery of plastics will contribute to the accumulation of approximately 1.2 × 10^13^ kg of plastic in soils and other environmental components [3].

It should be emphasized, however, that to date, soil contamination with these xenobiotics has received less attention from researchers than contamination of marine environments, despite the fact that their concentrations in soil may in some cases be up to 23 times higher than in water [4]. According to Ihenetu et al. [5,6], research on the contamination of agricultural soils by these xenobiotics is still in its infancy. Numerous critical questions, such as environmental risks and effective methods for removing this pool of contaminants, remain unanswered due to the inherent complexity of agricultural soils, which results from their variable physical, chemical, and biological composition. This is particularly alarming because agricultural soils represent one of the major sinks for microplastic accumulation in the environment. Its formation results from the gradual degradation of plastics. This process occurs as a result of long-term exposure to environmental factors such as mechanical abrasion, soil compaction, UV radiation, photolytic processes, and microbial activity [6,7,8]. Ultimately, by 2060, this will lead to a doubling or even tripling of microplastic concentrations in agriculturally used soils [9].

The scale of the problem in agricultural soils stems from practices such as plastic mulching [10], wastewater irrigation [10], and the use of sewage sludge [11]. Research by Gui et al. [12] also indicates that household compost in rural areas contains an average of 2400 microplastic particles per kg of dry weight of compost. Ultimately, between 6.3 × 10^4^ and 43 × 10^4^ tons of these contaminants enter soils in Europe and North America annually via compost [13]. At the root of this problem, however, lies the accumulation of polymers in soils and their transfer to organisms representing higher trophic levels through the food chain. This leads to the disruption of entire ecosystems. Importantly, these contaminants ultimately enter human food [14]. It is estimated that we consume approximately 0.2–6035 microplastic particles annually [15,16]. A link has been established between their presence and an increased risk of developing adenocarcinoma of the lung [17], stomach [18], and colon [19], as well as the induction of carcinogenic changes in kidney and liver cells [20].

Concerns about the effects of microplastic accumulation in ecosystems have led to a shift toward “green materials”, i.e., biodegradable alternatives synthesized from biopolymers derived from renewable sources. They require less energy during production while supporting global initiatives aimed at reducing plastic waste and mitigating climate change [21,22].

The most popular member of this group is polylactide (PLA) [23]. The first attempts to synthesize this compound, based on the condensation of lactic acid, are attributed to Théophile-Jules Pelouze (1845) [24]. PLA currently accounts for nearly half of the biodegradable plastics market (47%), with annual production estimated at approximately 300,000 tons [25,26]. Interest in this compound stems from the promising applications of PLA in sectors such as biomedicine, food packaging [27], and electronics, including innovative, multifunctional energy-harvesting systems, triboelectric nanogenerators [28], and systems capable of thermal and electromagnetic shielding [29]. Above all, the most important aspect is that it degrades under natural conditions, promoting ecological balance [30]. This material is valued for its lower carbon footprint compared to conventional plastics and for its ability to degrade under industrial composting conditions, leading to the formation of low-toxicity products [31,32]. Due to the presence of hydroxyl and carboxyl groups, lactic acid undergoes esterification, which leads to the formation of linear oligomers and cyclic lactide [33]. The second stage involves microorganism-mediated enzymatic hydrolysis to monomers, which are subsequently mineralized primarily to CO_2_ and H_2_O. However, hydrolysis occurs only after the polymers whose molecular weight decreases to 10,000–20,000 g mol^−1^ [34,35].

Low-density polyethylene (LDPE) was selected for the study because polyethylene (PE), along with polystyrene (PS) and polypropylene, is the source of microplastics that account for as much as 92.4% of all plastic waste [36]. LDPE is a low-density polyethylene produced by high-pressure polymerization of ethylene. It is chemically stable and thermally resistant up to approximately 80 °C [37]. Due to its transparency and flexibility, it is widely used in the production of plastic bags and packaging, for mulching agricultural land, and in construction [38,39]. It is also distinguished by its low production cost and energy efficiency [40]. Its hydrophobic backbone makes it an environmental challenge as a homochain polymer resistant to microbial degradation [41]. Nevertheless, recent reports indicate the biodegradation potential of bacteria such as *Bacillus* sp. AS3 and *Sphingobacterium* sp. AS8 against LDPE [42]. Similarly, according to Wanapat et al. [41], *Pseudomonas aeruginosa* KKU-LDPE4 can effectively promote the biodegradation of polyethylene. The list of microorganisms with biodegradation potential against LDPE is longer; nevertheless, species of the genus Pseudomonas are widely suggested: *Pseudomonas putida* [43] V, *Pseudomonas lundensis* [44], *Pseudomonas tolaasii* [45], and *Bacillus*: *Bacillus cereus* [46], *Bacillus licheniformis* [47], and *Bacillus safensis* [48].

The use of PLA and LDPE stems from their differing environmental properties. PLA is a bio-based polymer with biodegradation potential, whereas LDPE is a conventional, highly stable petrochemical polymer with high environmental durability, which allows for a comparison of two extremely different types of plastics. Furthermore, the different shapes of microplastics were also taken into account. The “shape difference” hypothesis suggests that microplastics with shapes different from soil particles may have a more significant impact on soil compared with spherical forms [49].

It should also be emphasized that microplastics lead to a loss of soil biodiversity by altering soil properties. Koskei et al. [50] demonstrated that exposure to microplastics reduces soil porosity and alters its pH [51]. In turn, Qi et al. [52] observed an increase in soil bulk density, which was interpreted as a tendency toward soil compaction. The presence of this pool of contaminants also adversely affects carbon, nitrogen, and phosphorus cycles, leading to a decline in soil fertility and thereby reducing its biological activity [53]. Another trend described is changes in the structure of bacterial communities in the soil, especially in microaggregates, and the formation of ecological niches of bacterial colonization defined as the “plastisphere”, which can also serve as a habitat for pathogens [36]. Within this, Yang et al. [54] identified bacteria with pathogenic potential, such as *Acinetobacter johnsonii* and *Escherichia coli*, indicating a potential increased health risk in soils contaminated with microplastics.

The research problem addressed is also linked to the need to develop effective strategies to mitigate the negative impacts of microplastics in agricultural ecosystems, which are currently the subject of multifaceted scientific debate on the international stage. A highly relevant bioremediation process is bioaugmentation, which supports the maintenance of biodiversity in agriculturally used soils and is strictly related to microbial biodegradation processes [55,56,57,58], as well as the application of humic acids, which are recognized as compounds improving soil health and stimulating microbial activity, thereby aligning with the principles of sustainable development [59].

*Pseudomonas umsongensis* is characterized by exceptionally high metabolic potential. A complete tph operon, responsible for the degradation of terephthalate to protocatechuate, has been identified in the strain’s genome, comprising the tphA1, tphA2, tphA3, tphB, and tphK genes. The strain also possesses a complete set of genes enabling the metabolism of ethylene glycol, including, among others, pedE, pedH, pedI, glcDEF, and aldB, leading to the conversion of this compound into central cellular metabolites. Of particular importance is the presence of numerous genes associated with lipid metabolism and *β*-oxidation, including enzymes such as 3-oxoacyl-ACP reductases, enoyl-ACP reductases, and 3-hydroxyacyl-CoA dehydrogenases, indicating a robust potential for the degradation of hydrophobic compounds [57].

There is an undeniable need for a detailed investigation of the effects of polymers with different degrees of biodegradability in agriculturally used soils, particularly in terms of their impact on bacterial communities and the potential risk of pathogen proliferation. This need arises from the lack of clearly identified, consistent patterns of soil bacterial responses to microplastics derived from PLA and LDPE mulch films, which constitutes an urgent research priority. Therefore, the aim of this study was to assess the effects of secondary microplastics derived from biodegradable polylactide (PLA) mulch film and conventional low-density polyethylene (LDPE) on the abundance, structure, and functional potential of the soil microbiome in agriculturally used soil, with particular emphasis on a potential increase in the risk associated with pathogenic bacteria. An innovative aspect of this study is the simultaneous evaluation of two soil remediation strategies applied after exposure to microplastics with contrasting chemical and environmental properties: biostimulation with humic acids and bioaugmentation with *Pseudomonas umsongensis*, a strain characterized by documented metabolic versatility toward polymer degradation products. Two research hypotheses were formulated in the study. The first hypothesis postulates that the presence of PLA and LDPE significantly modifies the structure and functional potential of the soil microbiome and increases the proportion of bacteria with pathogenic potential. The second hypothesis assumes that both humic acid biostimulation and *Pseudomonas umsongensis*-based bioaugmentation mitigate the negative effects of microplastic exposure, thereby supporting soil microbial regeneration.

## 2. Results

### 2.1. Effect of Soil Contamination with PLA and LDPE on the Abundance of Culturable Bacteria

**Figure 1:**
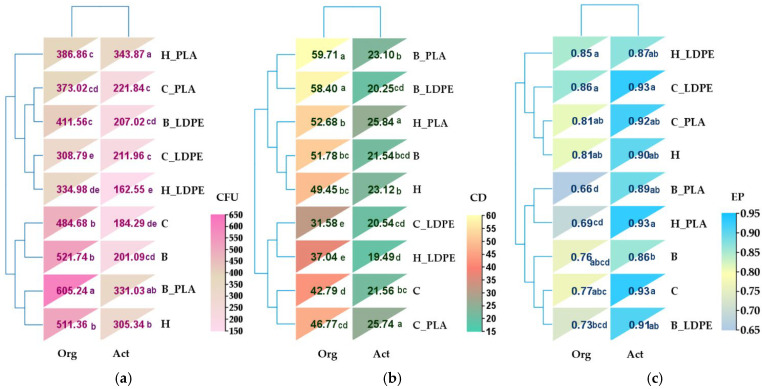
Heatmaps showing (**a**) the abundance of organotrophic bacteria (Org) and actinomycetes (Act) in soil (10^8^ CFU kg^−1^ d.m. of soil); (**b**) colony development (CD) indices of Org and Act; and (**c**) ecophysiological diversity (EP) indices of Org and Act. Color intensity corresponds to numerical values according to the scale. Homogeneous groups denoted with letters (a–e) were calculated separately for each microbial parameter (Tukey’s HSD test, *p* < 0.05). C—uncontaminated soil; PLA—soil contaminated with biodegradable mulch film; LDPE—soil contaminated with low-density polyethylene mulch film; B—soil bioaugmented with *Pseudomonas umsongensis*; H—soil biostimulated with humic acids.

Soil contamination with polylactic acid (PLA) mulch film and low-density polyethylene (LDPE) film significantly altered the abundance of organotrophic bacteria (Org) (Figure 1a). The magnitude of the negative impact caused by LDPE on this group of microorganisms exceeded that of PLA. The application of LDPE resulted in a 36.29% decrease in abundance, while PLA caused a 23.04% decrease compared to the control (C). The response of actinomycetes (Act) differed depending on exposure to PLA and LDPE. An increase in their abundance was observed in both PLA and LDPE samples, by 20.38% and 14.87%, respectively.

The implementation of preventive measures aimed to restore the soil’s microbial balance yielded unexpected results. Although a beneficial effect of humic acids (H) as well as bioaugmentation with *Pseudomonas umsongensis* (B) on the abundance of organotrophic bacteria and actinomycetes was observed in the control samples, the established homogeneous groups indicate a varied bioremediation potential of these treatments in mitigating the inhibitory effects of PLA and LDPE on organotrophic bacteria. In the case of organotrophic bacteria, a spectacular increase in abundance was recorded only after bioaugmentation (B) was applied in PLA-contaminated soil. The abundance of this group of microorganisms increased by up to 62.25% compared to the C_PLA plots. The response of actinomycetes to bioremediation treatments indicated that they enhanced the proliferation of this group of microorganisms with similar effectiveness, at 55% (H_PLA) and 49.22% (B_PLA). In the case of LDPE, significant effectiveness can be attributed only to the bioaugmentation of *Pseudomonas umsongensis* (B) against organotrophic bacteria.

The study also determined the effect of PLA and LDPE on the growth dynamics of Org and Act. Changes in this parameter were tracked based on the colony development index (CD) (Figure 1b). Analysis of the bacterial response to the applied microplastics through the lens of this index highlighted the stimulating effect of PLA and the inhibitory effect of LDPE on the growth of organotrophic bacteria. The application of LDPE caused a shift in their growth pattern from fast-growing to slow-growing, reducing the CD value by 26.20% (H_LDPE) compared to the control. Observation of the actinomycetes’ response indicated that, regardless of microplastic contamination, they are classified as slow-growing microorganisms. Furthermore, the effect of LDPE on this parameter was not as significant as that of organotrophic bacteria. After soil contamination with PLA mulch film, the growth dynamics of this group increased, reaching a CD value of 25.74 (C_PLA).

Importantly, each bioremediation method applied to soil contaminated with PLA and LDPE significantly increased the growth rate of organotrophic bacteria, whereas no such effect was observed for actinomycetes. In evaluating the effectiveness of these methods in mitigating the adverse effects of microplastics, particular attention should be paid to soil bioaugmentation with *Pseudomonas umsongensis* (B), which proved effective for both PLA and LDPE. The strength of the beneficial effect of this soil remediation method, as defined by the response of organotrophic bacteria, was reflected in 27.82% (B_PLA) and 84.93% (B_LDPE) increase in their abundance.

The interference of PLA and LDPE with the ecophysiological diversity of Org and Act, as determined by the EP index, was not as significant as that observed with CD (Figure 1c). The introduction of *Pseudomonas umsongensis* (B) into the soil caused selective changes in the structure of organotrophic bacteria in samples contaminated with both microplastics. A similar effect was observed in soil treated simultaneously with humic acids (H) and PLA. In this sample, the EP value was 14.81% lower than in C_PLA. In contrast to organotrophic bacteria, actinomycetes exhibited high ecophysiological stability.

### 2.2. Effect of Soil Contamination with PLA and LDPE on the Abundance and Diversity of Non-Culturable Bacteria

**Figure 2:**
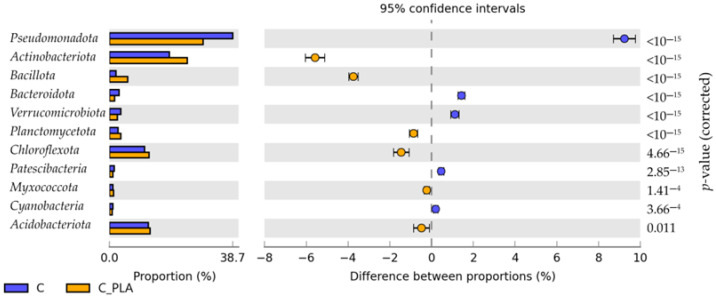

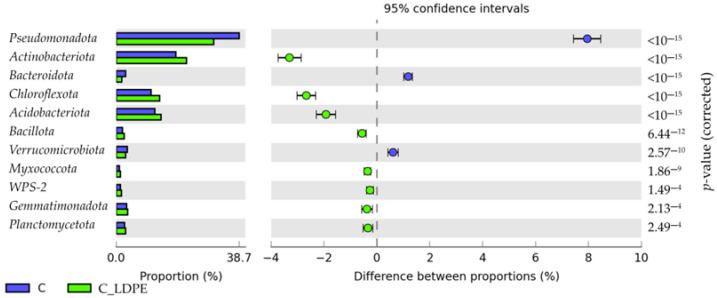
Effect of PLA and LDPE mulch film contamination on the relative abundance of dominant bacterial taxa (ASV ≥ 1%) in soil. C—uncontaminated soil; PLA—soil contaminated with biodegradable mulch film; LDPE—soil contaminated with low-density polyethylene mulch film.

16S rRNA gene amplicon sequencing of soil exposed to PLA and LDPE revealed differences in the structure of the bacterial community depending on the type of microplastic used (Figure 2). Specifically, at the phylum taxonomic level, bacteria representing *Pseudomonadota*, *Actinomycetota*, *Acidobacteriota*, and *Chloroflexota* dominated in all samples, accounting for 34.70%, 22.03%, 11.64%, and 11.09%, respectively. To facilitate interpretation, 13 taxa were defined in the experiment, focusing on taxa representing more than 1% of total assigned amplicon sequence variants (ASVs). The presence of specific compounds then generated rearrangements within representative phylum pools. Notably, exposure to PLA contributed to an increase in the abundance of only three taxa, *Bacillota* (87.95%), *Planctomycetota* (12.91%), and *Actinobacteriota* (10.55%), compared to the control. Conversely, a negative effect of PLA was observed for the following ten taxa, as evidenced by a decrease in the number of ASVs in each phylum: *Bacteroidota* (56.00%), *Patescibacteria* (41.79%), *Verrucomicrobiota* (41.69%), *Pseudomonadota* (35.11%), *Cyanobacteria* (30.87%), *WPS-2* (16.76%), *Acidobacteriota* (11.35%), *Gemmatimonadota* (10.80%), *Myxococcota* (5.07%), and *Chloroflexota* (3.50%). As a result, the abundance of ASVs across all phyla was reduced, dropping from 65,816 ASVs in uncontaminated soil (C) to 56,120 ASVs in PLA-contaminated soil (C_PLA). Finally, it is worth noting that the proportion of *Bacillota* and *Actinomycetota* representatives in PLA-contaminated soil was higher than in the control soil by 3.74% and 5.58%, respectively, whereas that of *Pseudomonadota* decreased by 9.24%.

A different trend was observed in soil contaminated with LDPE. First and foremost, the ASV count increased to 67,271 (Figure 2), indicating a beneficial effect on the proliferation of bacteria belonging to nine phyla, while negatively affecting only four taxa. Marked increases of 38.86%, 31.16%, 27.00%, 21.03%, and 20.15% were recorded for the phyla *Myxococcota*, *Bacillota*, *Chloroflexota*, *WPS-2*, and *Actinomycetota*. In contrast, negative effects of LDPE were observed for *Bacteroidota*, *Pseudomonadota*, and *Verrucomicrobiota*, reducing their abundance by 38.67%, 18.81%, and 15.50%, respectively, compared to the control. 

**Figure 3:**
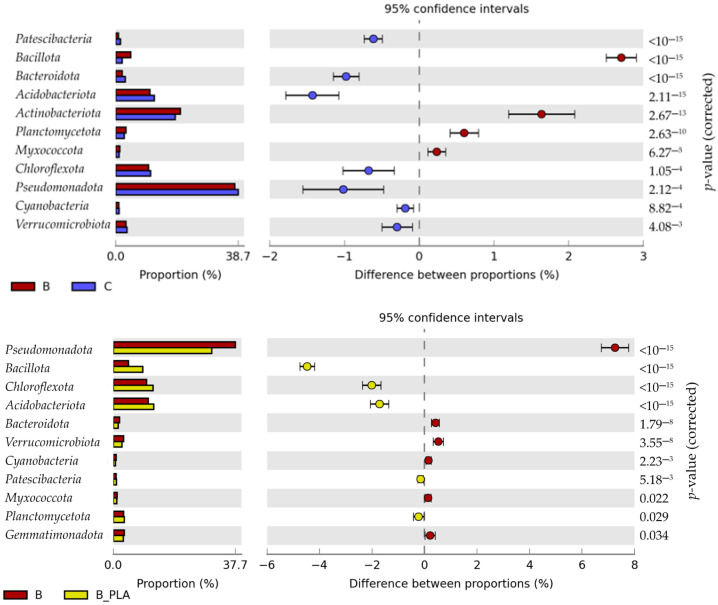

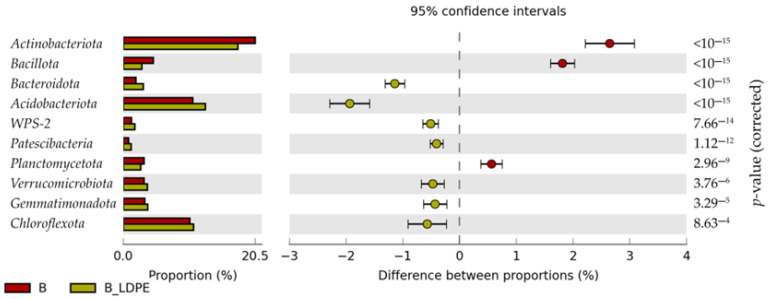
Effect of PLA and LDPE mulch film contamination and bioaugmentation with *Pseudomonas umsongensis* on the relative abundance of dominant bacterial taxa (ASV ≥ 1%) in soil. C—uncontaminated soil; PLA—soil contaminated with biodegradable mulch film; LDPE—soil contaminated with low-density polyethylene mulch film; B—soil bioaugmented with *Pseudomonas umsongensis*.

Given the potential of the bioremediation methods employed, it was demonstrated that bioaugmentation with *Pseudomonas umsongensis* in combination with PLA enhanced the proliferation of representatives of all identified phyla, with a total ASV value 16.84% higher than that at the C_PLA object (Figure 3). This trend was likely influenced by a significant stimulation of the proliferation of bacteria belonging to the phylum *Bacillota* (by as much as 92.31%), *Verrucomicrobiota* (35.10%), and *Pseudomonadota* (24.19%) in PLA-contaminated soil. Lower bioaugmentation efficacy was observed in soil exposed to LDPE.

**Figure 4:**
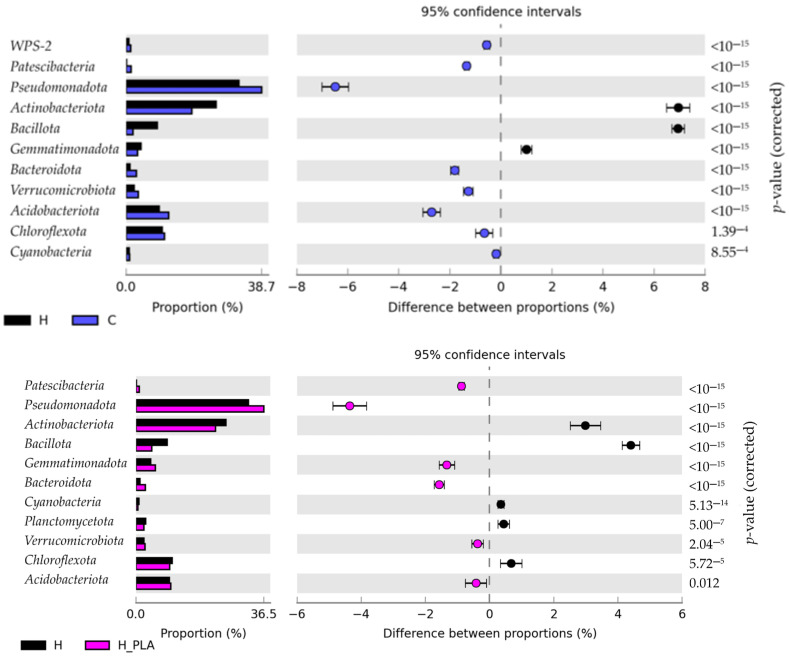

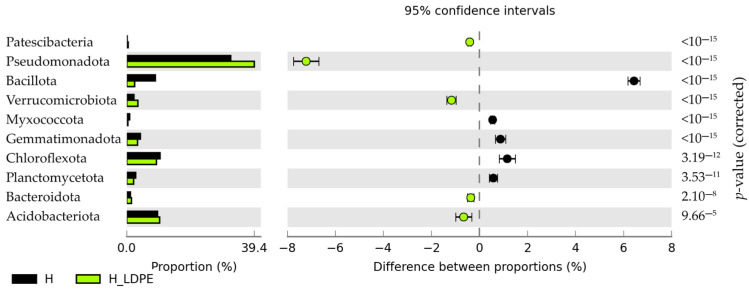
Effect of PLA and LDPE mulch film contamination and biostimulation with humic acids on the relative abundance of dominant bacterial taxa (ASV ≥ 1%) in soil. C—uncontaminated soil; PLA—soil contaminated with biodegradable mulch film; LDPE—soil contaminated with low-density polyethylene mulch film; H—soil biostimulated with humic acids.

The humic acids (H) used did not have such a positive effect on bacterial re-assembly at this taxonomic level (Figure 4). In the presence of PLA, growth inhibition of bacteria from seven phyla was observed, along with a marked increase in the abundance of *Bacteroidota* by as much as 95.45%, *Gemmatimonadota* by 80.14%, and *Pseudomonadota* by 36.13% compared to the C_PLA control. Bacteria of most taxa showed sensitivity to the combination of humic acids and LDPE, which had a beneficial effect only on the abundance of *Actinomycetota*, *Acidobacteriota*, and *Verrucomicrobiota*.

**Figure 5:**
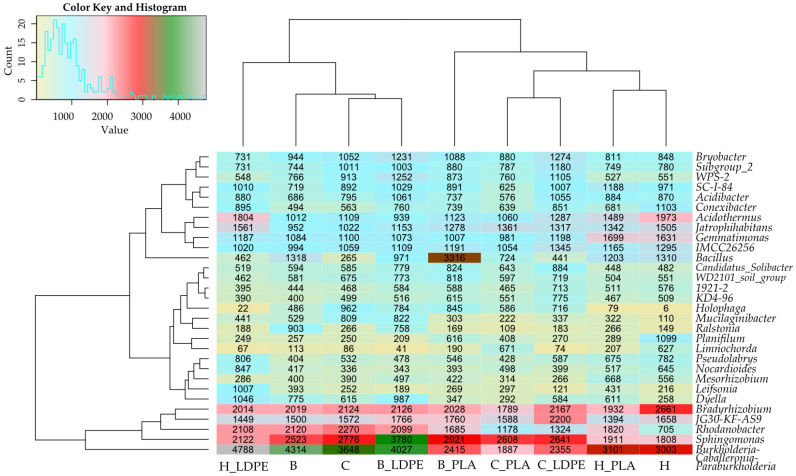
Dominant bacterial genera in soil contaminated with PLA and LDPE presented in a heatmap. The color gradient in the “Color Key and Histogram” panel corresponds to ASV ≥ 1% values of the analyzed bacterial genera. The blue line represents the data distribution histogram. Dendrograms group samples and bacterial genera based on similarity of profiles. C—uncontaminated soil; PLA—soil contaminated with biodegradable mulch film; LDPE—soil contaminated with low-density polyethylene mulch film; B—soil bioaugmented with *Pseudomonas umsongensis*; H—soil biostimulated with humic acids.

Changes in bacterial structure observed at the phylum taxonomic level resulted in corresponding reclassifications at the genus level (Figure 5). Consequently, the response of microorganisms to PLA and LDPE also differed. Of the 29 identified genera, 19 were found to be sensitive, while 10 were stimulated to proliferate in PLA-contaminated soil. The ASV abundance of this treatment was dominated by the genera *Sphingomonas* (2608 ASV), *Burkholderia-Caballeronia-Paraburkholderia* (1887 ASV), *Bradyrhizobium* (1789 ASV), *JG30-KF-A59* (1588 ASV), and *Jatrophihabitans* (1361 ASV), with each accounting for over 5% of the total and comprising 10.61%, 7.68%, 7.28%, 6.46%, and 5.54%. Nevertheless, the abundance of *Burkholderia-Caballeronia-Paraburkholderia*, *Bradyrhizobium*, and *Sphingomonas*, assigned to the phylum *Pseudomonadota*, decreased by 48.27%, 15.77%, and 6.05% compared to the control. It is also worth noting the 2.7-fold increase in the number of ASVs of the genus *Bacillus* and the 7-fold increase in the abundance of the genus *Limnochorda*, representatives of *Bacillota*, in this study. The application of LDPE inhibited the proliferation of only 9 bacterial genera, while stimulating the growth of the remaining 20 compared to the control. The highest abundance of ASVs was recorded for *Sphingomonas* (2641 ASVs), *Burkholderia-Caballeronia-Paraburkholderia* (2355 ASVs), *Bradyrhizobium* (2167 ASVs), and *JG30-KF-A59* (2200 ASVs). This abundance was higher than in PLA-contaminated treatments.

A beneficial modulation of bacterial community structure at the genus level was achieved through bioaugmentation with *Pseudomonas umsongensis* (B), particularly in PLA-contaminated soil (Figure 5). Within this group of treatments, stimulation of the proliferation of all identified bacterial genera was observed, except for *Rhodanobacter*, *Jatrophihabitans*, *Nocardioides*, *Leifsonia*, and *Limnochorda* relative to the C_PLA treatment. It is worth noting that the abundance of the genus *Bacillus* increased from 724 ASV to 3316 ASV. The high effectiveness of this method was also observed in LDPE-contaminated soil. However, it should be emphasized that the increase in ASV abundance of all bacterial genera from 28,885 (B) to 33,139 (B_LDPE) was associated with the proliferation of the representative genera *Burkholderia-Caballeronia-Paraburkholderia* (4027), *Sphingomonas* (3780), and *Rhodanobacter* (2099), which together accounted for 36.31% of the microbial community. In their case, an increase in relative abundance of 71.00%, 58.53%, and 43.13%, respectively, was observed compared to soil subjected to LDPE stress. Also noteworthy is the spectacular increase in bacteria of the genera *Dyella*, *Ralstonia*, representatives of the phylum *Pseudomonadota*, and *Bacillus*—*Bacillota*, within this group of organisms. After analyzing the bacterial response to the co-occurrence of H and individual microplastics in the soil, it was found that regardless of whether PLA or LDPE was applied to the soil, the abundance of the following 10 bacterial genera increased: *Burkholderia-Caballeronia-Paraburkholderia*, *Rhodanobacter*, *Acidothermus*, *Mucilaginibacter*, *Dyella*, *Pseudolabrys*, *Mesorhizobium*, *Ralstonia*, *Bacillus* and *Leifsonia.* The only difference was in the strength of stimulation for individual genera. In PLA-treated soil, the magnitude of the spectacular effect of humic acids (H) was 115.04% for the genus *Ralstonia* and 87.47% for *Mesorhizobium*. In turn, in LDPE-contaminated soil enriched with H, a 7-fold increase in the number of ASVs of the genus *Leifsonia* was observed, along with a 98.84% increase in the abundance of *Burkholderia-Caballeronia-Paraburkholderia*. It is worth noting that for the genus *Dyella*, the extent of HA’s beneficial effect was equally significant in soil contaminated with PLA (84.39%) as in soil contaminated with LDPE (75.17%). Importantly, in both samples (H_PLA and H_LDPE), there was an abundance of the genera *Holophaga*, *WPS_2*, and *Candidatus_Solibacter*; in the H_PLA sample, the genus *Limnochorda* decreased by as much as 71.28% compared to C_PLA; and in the H_LDPE sample, the genera *JG30-KF-AS9* (35.58%), *Bryobacter* (43.88%), 1921-2 (45.82%), and *KD4-96* (50.78%) decreased by 71.28% in the H_PLA sample compared to C_PLA.

**Figure 6:**
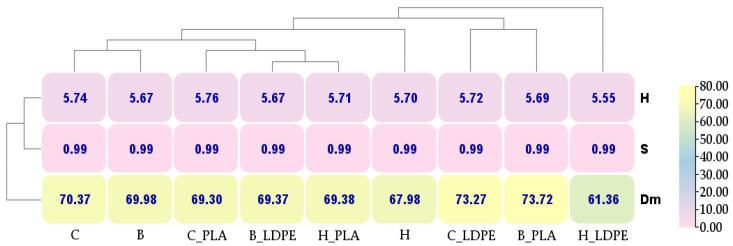
The heatmap presents bacterial diversity indices in soil contaminated with PLA and LDPE. The values were row-standardized, and the colors represent relative levels of the analyzed indices. Dendrograms show hierarchical clustering based on similarity of samples and diversity indices. H’—Shannon–Wiener; S—Simpson, Dm—Margalef. C—uncontaminated soil; PLA—soil contaminated with biodegradable mulch film; LDPE—soil contaminated with low-density polyethylene mulch film; B—soil bioaugmented with *Pseudomonas umsongensis*; H—soil biostimulated with humic acids.

Changes in bacterial composition at the genus level were also assessed using taxonomic diversity indices (Figure 6). After adding PLA mulch film to the soil, a slight decrease in the richness of this taxon pool was observed, whereas in soil with LDPE, the Margalef index (Dm) increased by 4.14% compared to the control. The Shannon–Wiener index (H’) showed no significant changes, indicating the stability of overall bacterial diversity at the genus level. Bioaugmentation with *Pseudomonas umsongensis* in PLA-contaminated soil clearly increased the value of this parameter (H’) and the Margalef index (Dm). In LDPE-contaminated soil, however, both after bioaugmentation (B) and after the application of humic acids (H), a 17.01% decrease in the index value was observed compared to the LDPE-contaminated control (C_LDPE), suggesting that in the presence of microplastics that are difficult to degrade, preventive measures may limit genus-level diversity by favoring a few dominant taxa.

**Figure 7:**
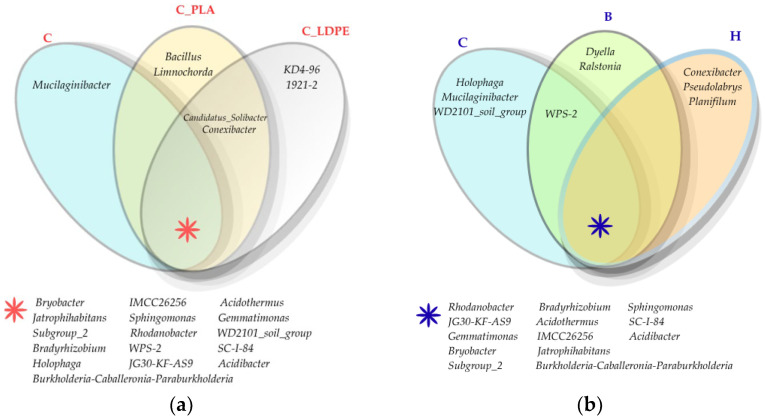
Unique and common bacterial species in soil contaminated with PLA and LDPE (**a**) and subjected to biostimulation (**b**); C—uncontaminated soil; PLA—soil contaminated with PLA mulch film; LDPE—soil contaminated with low-density polyethylene (LDPE) mulch film; B—soil bioaugmented with *Pseudomonas umsongensis*; H—soil biostimulated with humic acids.

Changes in bacterial composition identified at the genus level reflect two trends observed in the study (Figure 7a). The first trend indicates a group of sixteen native genera identified in both control object and those exposed to PLA and LDPE. The second trend revealed the genera *Bacillus* and *Limnochorda* as taxa unique to PLA-contaminated soil. In LDPE-treated object, the genera *KD4-96* and *1921-2* were identified as unique. It should also be emphasized that bioaugmentation with *Pseudomonas umsongensis* (B) and soil biostimulation with humic acids (H) influenced bacterial community composition (Figure 7b). The inoculum used (B) enhanced the proliferation of *Dyella* and *Ralstonia*, which were unidentified in the residual samples, while humic acids (H) stimulated the growth of bacteria from the genera *Conexibacter*, *Pseudolabrys*, and *Planifilum*.

### 2.3. Predicted Metabolic and Ecological Functions of Bacterial Communities

**Figure 8:**
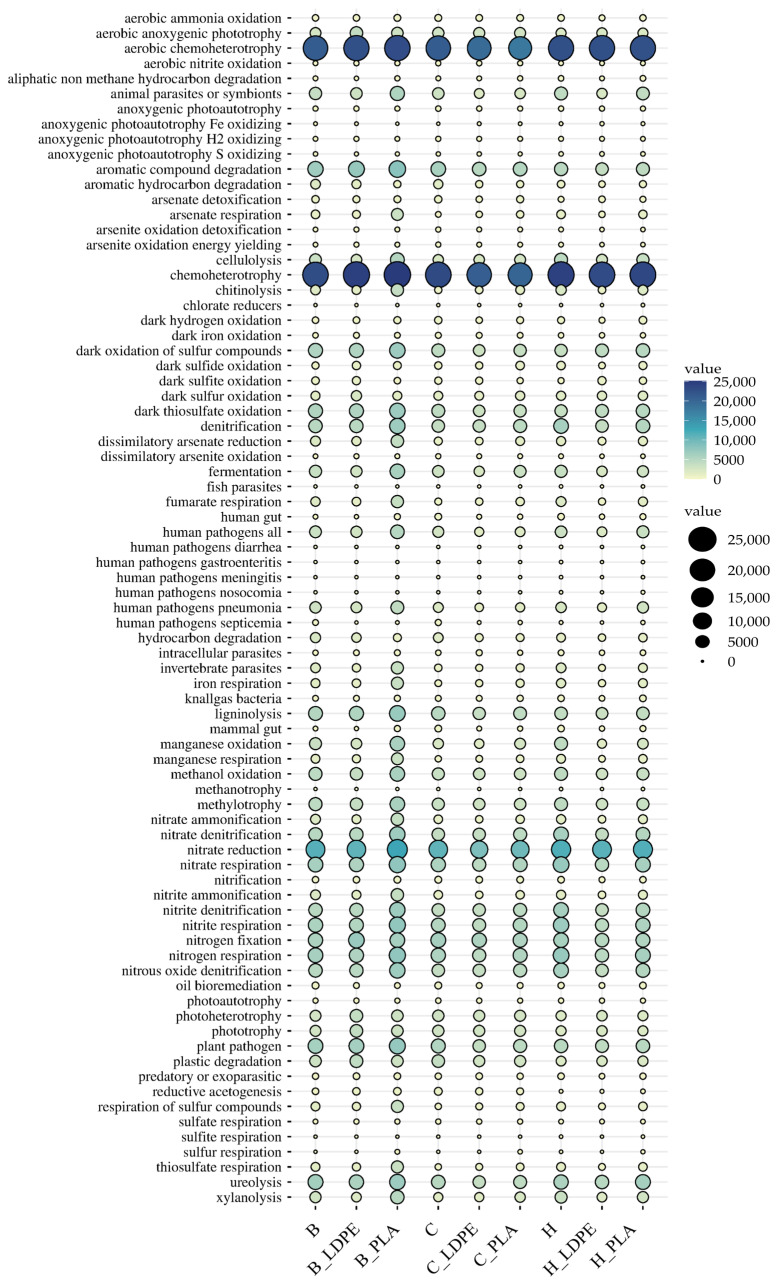
Predicted metabolic and ecological functions of bacterial communities based on 16S rRNA gene sequencing—ASV. Functional annotations were inferred using FAPROTAX and expressed as the number of sequence reads assigned to functional groups. C—uncontaminated soil; PLA—soil contaminated with biodegradable mulch film; LDPE—soil contaminated with low-density polyethylene mulch film; B—soil bioaugmented with *Pseudomonas umsongensis*; H—soil biostimulated with humic acids.

Analysis of the results using FAPROTAX revealed trends in the predicted metabolic and ecological functions of bacteria across all soil samples (Figure 8). The dominant functional groups were associated with the following processes: nitrate reduction > nitrate respiration > nitrogen fixation > aromatic compound degradation > nitrite respiration > ureolysis > nitrate denitrification. Plant taxa associated with pathogenic potential also constituted a significant community. The application of PLA and LDPE polymers to the soil induced changes in the predicted functional profile of the bacteriobiome. PLA had a beneficial effect on bacterial proliferation with the functions listed above, whereas exposure to LDPE reduced their proportion. PLA increased the abundance of bacteria associated with the functions listed above, whereas exposure to LDPE reduced their abundance. Humic acids (H) effectively stimulated the proliferation of bacteria exposed to PLA; however, this effect was not observed for microorganisms involved in nitrogen cycle processes, including nitrate respiration, nitrite respiration, nitrogen fixation, and nitrogen respiration, in soil contaminated with LDPE.

**Figure 9:**
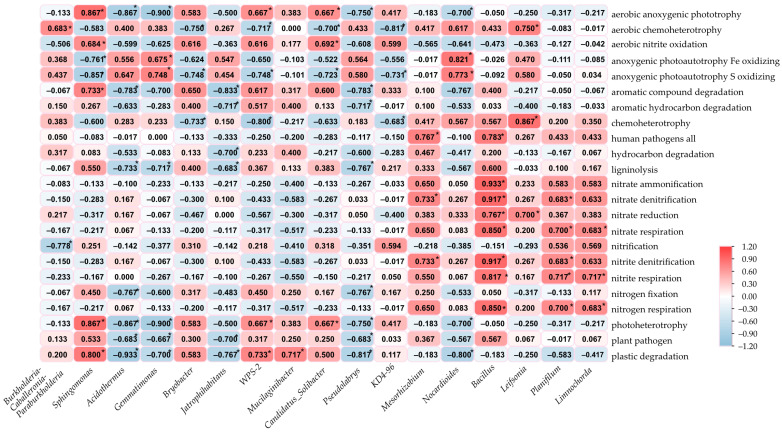
Heat map showing Spearman’s rank correlations between representative bacterial genera in soil and predicted metabolic and ecological functions. The color scale represents correlation coefficients, and statistically significant correlations (*p* < 0.05) are marked with an asterisk (*).

Analysis of Spearman’s rank correlation coefficients between representative soil genera and bacterial predicted functional profiles revealed significant relationships (Figure 9). A strong positive correlation was found between chemoheterotrophy and the genera *Leifsonia* (r = 0.867) and *Burkholderia–Caballeronia–Paraburkholderia* (r = 0.683). Metabolic processes related to nitrogen transformation were primarily driven by bacteria of the genera *Bacillus*, *Leifsonia*, *Planifilum*, *Limnochorda*, and *Mesorhizobium*. Taxa showing significant correlations with plastic degradation potential were also identified. The strongest positive correlations were observed for the genera *Sphingomonas* (r = 0.800), *WPS-2* (r = 0.733), and *Mucilaginibacter* (r = 0.717). At the same time, negative correlations were found between the analyzed function and taxa such as *Acidothermus*, *Gemmatimonas*, *Jatrophihabitans*, *Pseudolabrys*, and *Nocardioides*. It is worth noting that *Sphingomonas* also exhibited a high correlation coefficient (r = 0.733) for the degradation of aromatic compounds. Spearman’s rank correlation analysis also revealed positive associations between taxa associated with pathogenic potential-related functions and taxa from the genera *Sphingomonas* and *Bacillus* (potential plant pathogens), as well as *Bacillus* and *Mesorhizobium* (potential human pathogens).

**Figure 10:**
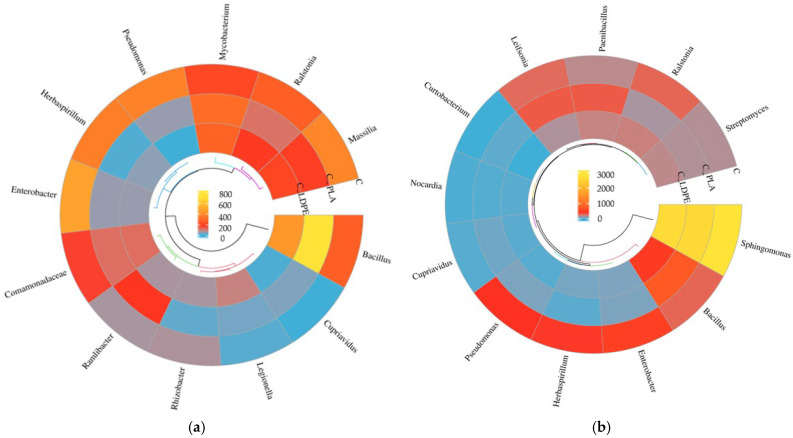
Heatmap showing taxa with predicted pathogenic potential for humans (**a**) and plants (**b**) in soil contaminated with PLA and LDPE. The values represent the number of sequencing reads assigned to taxa with predicted pathogenic potential (16S rRNA–ASV) based on FAPROTAX functional annotation. C—uncontaminated soil; PLA—soil contaminated with biodegradable mulch film; LDPE—soil contaminated with low-density polyethylene mulch film; B—soil bioaugmented with *Pseudomonas umsongensis*; H—soil biostimulated with humic acids.

FAPROTAX analysis indicated the presence of taxa assigned to functional groups associated with potential pathogenicity in soil (Figure 10). Six genera, *Herbaspirillum*, *Ralstonia*, *Pseudomonas*, *Enterobacter*, *Cupriavidus*, and *Bacillus*, were common to both groups. *Enterobacter* (479 ASV) and *Herbaspirillum* (358 ASV) were the most abundant in the control samples. It is noteworthy that soil contamination with both PLA and LDPE resulted in a reduction in their abundance by as much as 90.19% and 95.25% (PLA) and 89.98% and 88.55% (LDPE), respectively, compared to the control. A different trend was also observed. The polymers enhanced the proliferation of the genus *Bacillus*. Among genera classified as potentially human-associated pathogens, *Mycobacterium* abundance also increased (Figure 10a). PLA had a stronger stimulating effect on this parameter (an increase of 46.41%) than LDPE (an increase of 33.01%). The analyzed contaminants altered the composition of plant taxa associated with pathogenic potential (Figure 10b). The most abundant bacterial genus across all samples was *Sphingomonas*, whose abundance decreased from 2776 ASVs in the control (C) to 2608 and 2641 ASVs in the PLA and LDPE treatments, respectively. Different responses to polymers were observed for *Leifsonia* and *Curtobacterium*. PLA had a beneficial effect on their proliferation, while LDPE inhibited it. The abundance of *Leifsonia* decreased by 48.02%, whereas *Curtobacterium* was detected only in soil exposed to PLA.

## 3. Discussion

### 3.1. Effects of Soil Contamination with PLA and LDPE and Biostimulation with Humic Acids and Pseudomonas umsongensis on Culturable Bacteria

Our research demonstrated that organotrophic bacteria are sensitive to soil contamination with both PLA and LDPE. The negative impact of the tested polymers was 36.29% (PLA) and 23.04% (LDPE) (Figure 1). Analysis of the response of organotrophic bacteria is justified, as these microorganisms form complex communities and participate in multifaceted interactions [60]. Deciphering these interactions is important because there is no consensus on the impact of microplastics on soil bacteria. The adverse effects observed may be attributed to the generation of reactive oxygen species [61], additive leaching [62], and changes in water retention in microplastic-contaminated soil [63]. Gao et al. [64] also demonstrated that in soil containing PLA, the ratio of Gram-positive to Gram-negative bacteria shifted, accompanied by an increase in the proportion of saturated fatty acids in PLFA profiles, interpreted as a sign of environmental stress. At the same time, the abundance of iso- and anteiso-fatty acids decreased, indicating reduced microbial biomass, including Gram-positive bacteria. These changes ultimately led to disruptions in the functioning of organotrophic bacteria and a decrease in the intensity of organic matter decomposition processes [65].

A significant trend observed in our research was an increase in the abundance of actinomycetes exposed to both PLA and LDPE. We also observed greater stimulation of the proliferation of this group of microorganisms in soil contaminated with PLA than with LDPE. These relationships can be explained in several ways. First and foremost, they stem from the biodegradation potential of actinomycetes associated with biosurfactant production. As with bacteria, a wide range of enzymes, primarily hydrolases, may play a significant role, including lipases (EC 3.1.1.3), esterases (EC 3.1.1.x) and cutinases (EC 3.1.1.74), as well as serine proteases (EC 3.4.21) responsible for PLA degradation [66,67]. Among actinomycetes, significant producers of these enzymes include the genera *Actinomadura*, *Thermopolyspora*, *Saccharothrix*, and *Amylocatopsis* [68,69]. It is worth noting that PLA degradation mediated by serine proteases attributed to *Amylocatopsis* proceeds via a two-step process. Amino acids located in the active site of enzymes (serine, histidine, and aspartic acid) play a significant role in enzymatic catalysis. In the second stage of PLA degradation, PLAase I–III enzymes depolymerize oligomers formed in the first stage of the process [70]. Enzymes effective in the degradation of low-density polyethylene (LDPE), a polymer resistant to this process, include laccases and peroxidases. These enzymes, which belong to the oxidoreductase class, are produced by actinomycetes of the genera *Rhodococcus* and *Streptomyces*, such as *Streptomyces coelicoflavus* and *Streptomyces gougerotti* [69]. The ability of actinomycetes to transform polymers also stems from their dominance in the late stages of organic matter decomposition [71], which to some extent corresponds to their definition as slow-growing microorganisms, as demonstrated in our experiment.

The study also evaluated the effectiveness of preventive measures based on bioaugmentation with the *Pseudomonas umsongensis* strain (B) and biostimulation with humic acids (H). Our findings indicate that bioaugmentation is a more effective method for bioremediating microplastic-contaminated soil than biostimulation. This is supported by a spectacular 62.25% increase in the abundance of organotrophic bacteria in the test samples (B_PLA), the induction of growth in the abundance of this group of bacteria in LDPE-contaminated soil, and the enhancement of their proliferation dynamics, which ranged from 27.82% (B_PLA) and 84.93% (B_LDPE). The expectation of a beneficial effect of the *Pseudomonas umsongensis* strain was supported by reports from other researchers [72,73]. The results indicate that bacteria of the genus *Pseudomonas* colonizes the biologically accessible surface of microplastics and form a biofilm [72]. It has also been demonstrated that this potential is underpinned by the presence of genes (algD, AlgA, algC) of the alginate biosynthesis pathway, a polymeric substance that ensures the structural integrity of the organized bacterial community [73]. The EPS matrix facilitates cell adhesion to hydrophobic microplastic surfaces and increases resistance to fluctuations in moisture and nutrient availability, thereby promoting the maintenance of stable colonization of the microhabitat [74]. Consequently, the biofilm may act as a structure sustaining an active population of the strain in the immediate vicinity of the substrate, which distinguishes bioaugmentation from humic acid biostimulation. *Pseudomonas umsongensis* GO16 also exhibits high metabolic versatility associated, among others, with the presence of catabolic plasmids (e.g., pENK22), which may modify the pool of available carbon compounds and consequently influence the structure of other taxa [57]. Salinas et al. [56] demonstrated that *Pseudomonas putida* was responsible for the biodegradation of LDPE–LLDPE (low-density polyethylene with a linear chain structure and short side branches). Liu et al. [75] demonstrated increased efficiency of processes involved in retention and mobility of microplastic particles in biofilms formed by *Pseudomonas aeruginosa* and *Pseudomonas putida*; these processes were controlled using the pLac-yedQ and pBAD-yhjH genetic systems, which regulate biofilm structure.

### 3.2. Effects of Soil Contamination with PLA and LDPE and Biostimulation with Humic Acids and Pseudomonas umsongensis on Non-Culturable Bacteria

In the present study, it was demonstrated that PLA and LDPE applied to soil interfere with the structure of soil bacterial communities. The results clearly indicate that the type of material is a key factor in determining the direction of these changes, which is consistent with the proposed research hypothesis. Although representatives of the phyla *Pseudomonadota*, *Actinomycetota*, *Acidobacteriota*, and *Chloroflexota* were dominant in all samples, PLA promoted *Actinomycetota*, whereas LDPE promoted *Actinobacteriota* and *Chloroflexota* (Figure 2). PLA also reduced ASV abundance across as many as nine phyla, while LDPE reduced it in only three. The observed trends likely result from the low degradability of LDPE, which promotes the stabilization and growth of microbial biofilms [76]. In contrast, PLA, which undergoes partial hydrolysis, forms low-molecular-weight products that increase substrate availability, thereby generating more selective environmental pressure [77].

Notably, exposure to PLA, similarly to LDPE, contributed to an increase in the abundance of *Bacillota* taxa by 87.95% and 31.16%, respectively. This was most likely due to the fact that the applied polymers stimulated the proliferation of the genus *Bacillus*, belonging to this phylum. PLA increased *Bacillus* abundance approximately 2.7-fold, whereas LDPE increased it by 66.42%. The obtained results, indicating a selective response of *Bacillus* to the presence of microplastics, are consistent with reports from other studies [78,79,80,81]. According to Yang [78], *Bacillus* led to a 50% reduction in the tensile strength of PE film, while *Bacillus siamensis* was able to degrade LDPE by 8.46% [79]. In the studies by Sadler and Wallace [80], it was also demonstrated that *Bacillus subtilis*, after transformation with a plasmid encoding phosphopantetheinyl synthetase (Sfp), acquired the ability to biotransform microplastic degradation products into aromatic compounds, including vanillin. Therefore, polyethylene (PE) degradation should also be considered. Under aerobic conditions, this process proceeds in stages, involving the conversion of the polymer into alkanes, which are subsequently oxidized by cytochrome P450 to alcohols, and by alcohol dehydrogenase to carbonyl compounds and acids. Subsequent steps, involving Baeyer–Villiger monooxygenase and esterases, lead to the formation of metabolites that enter central metabolic pathways. However, PE biodegradation is characterized by limited efficiency in soil [81,82].

Differences in bacterial responses to soil contamination with PLA and LDPE were also observed based on changes in the ASV abundance of the phylum *WPS-2* (Figure 2). Exposure to PLA resulted in a 16.76% inhibition of *WPS-2* taxa proliferation compared to the control, whereas this parameter increased by 21.03% in LDPE-contaminated soil. The inhibitory effect of microplastics on microorganisms assigned to this phylum, as well as a weakening of their connectivity within the microbial network structure, was also reported by Huang et al. [67]. The increase in *WPS-2* ASV abundance in the presence of LDPE can be interpreted as a consequence of the formation of new colonization niches and the associated selective pressure favoring oligotrophic bacteria, which are capable of functioning under conditions of limited carbon availability and a changing structure of microbial competition [83,84]. This trend may be related to emerging first reports on microorganisms assigned to *WPS-2*, indicating their ability to utilize hydrogen and carbon dioxide as metabolic substrates in harsh Antarctic ecosystems [85].

Bioaugmentation with *Pseudomonas umsongensis* (B) can be considered a more effective method for increasing bacterial richness, microbial interactions, and ultimately improving soil fertility, regardless of the contaminant applied, which remains consistent with the proposed research hypothesis. Nevertheless, both bioaugmentation and humic acid biostimulation mitigated the negative impact of PLA on the proliferation of bacteria belonging to the phylum *Pseudomonadota* (Figure 3 and Figure 4). In the B_PLA and H_PLA treatments, an increase of 24.19% and 36.13%, respectively, in the abundance of this phylum was observed compared with soils contaminated with this microplastic. Such a beneficial effect of *Pseudomonas umsongensis* bioaugmentation was reflected in a significant increase in the abundance of *Bacillus* sp., whereas humic acids promoted *Ralstonia* sp. (by up to 111.05%), belonging to the phylum *Pseudomonadota*.

The observed response of these bacteria is consistent with the findings of Biki et al. [86]. The authors demonstrated that *Ralstonia* sp. SKM2 and *Bacillus* sp. SM1 exhibit the ability to biodegrade not only PLA but also the recalcitrant polymer LDPE. The mass loss of the film, associated with observed changes in polymer chemical structure, including alkane, ether, and alcohol bonds, was 39.2% for *Ralstonia* sp. and 18.9% for *Bacillus* sp. In addition, Cho and Cho [87] showed that functional gene prediction analysis identified genes K01126 (glycerophosphodiester phosphodiesterase) and K01048 (lysophospholipase) as associated with high PLA degradation efficiency in *Ralstonia* sp. AF1 strain.

The combined application of humic acids and LDPE resulted in a higher abundance of *Actinobacteriota*, which was associated with a sevenfold increase in ASV abundance assigned to the genus *Leifsonia* (1007). The observed relationships may be explained by the ability of these organisms to biosynthesize extracellular cellulose, a process catalyzed by the cellulose synthase complex (CelA/CelS-like proteins). This, in turn, promotes the stabilization of soil microaggregates [88]. The complex structure of humic acids, characterized by a high abundance of functional groups such as carboxyl, phenolic, and quinone groups, also provided an abundant source of carbon and energy for these bacteria [56]. Humic acids exhibit multifunctional environmental effects, including redox properties, modification of microplastic surfaces, and stimulation of microbial adhesion and activity, leading to non-specific stimulation of native microbial communities [89]. In contrast to bioaugmentation, this effect depends on the activity and structure of the autochthonous microbiome, which may limit its bioremediation efficiency.

### 3.3. Metabolic- and Ecological-Based Characterization of Taxa Associated with Pathogenic Potential

Soil contamination with polymers did not merely modify but strongly reshaped the structure of potential pathogenic bacteria (Figure 9 and Figure 10). In soils contaminated with both PLA and LDPE, a decrease in the abundance of *Enterobacter* and *Herbaspirillum* was observed, along with stimulation of *Bacillus* proliferation. *Bacillus* genus comprises both plant and human taxa associated with pathogenic potential. Among human potential pathogens, an increased abundance of *Mycobacterium* was observed, with a more pronounced stimulatory effect for PLA. Among plant potential pathogens, both polymers exerted an inhibitory effect on *Sphingomonas*. Bacteria of the genus *Curtobacterium* were detected exclusively in PLA-exposed treatments.

The plastisphere may constitute a unique microbial niche that promotes the colonization and persistence of pathogenic microorganisms, potentially enhancing their ecological impact through the upregulation of functional pathways associated with virulence and pathogenicity [90]. The response of potential pathogenic taxa to the presence of PLA and LDPE observed in the present study is in agreement with previous reports [91,92,93,94,95,96]. The genus *Enterobacter* is considered a clinically and environmentally relevant taxon. Huang et al. [91] demonstrated that the abundance of *Enterobacter bugandensis* in soil exposed to a biodegradable polymer decreased by up to 80%, whereas no reduction in comparable magnitude was observed in polyethylene (PE)-amended soil. Likewise, the microplastic-associated niche was not conducive to the proliferation of *Herbaspirillum* sp. [92]. In contrast, Ma et al. [93] reported that polyethylene surfaces were predominantly colonized by bacterial communities exhibiting high metabolic potential, with the genus *Mycobacterium* being the dominant taxon.

Similarly, Muangchinda and Pinyakong [94] demonstrated that bacteria of the genus *Mycobacterium* constituted up to 27% of the bacterial consortium detected on the surface of LDPE, and Munhoz et al. [95] showed that *Mycobacterium* can facilitate the degradation of this polymer in compost. It is also worth noting the presence of bacteria of the genus *Curtobacterium* in PLA-exposed soil, a potential plant pathogen belonging to relatively poorly studied taxa. However, Mijatović Scouten et al. [96] indicate that *Curtobacterium aetherium*, comprising strains originating both from agricultural millet (G77) and the stratosphere (L6-1), is characterized by a hybrid origin. The stratospheric strain is associated with increased resistance to desiccation and UV radiation, which may confer tolerance to environmental stress factors and promote its proliferation on the surface of PLA.

### 3.4. Limitations of the Study

The results of this study should be interpreted in relation to the applied experimental model and its design assumptions, which define the scope of interpretative limitations. The experiment was conducted under controlled conditions, using a relatively high dose of microplastics to reflect strong environmental pressure. The experimental setup included a soil–plant system without a plant-free control; therefore, the observed effects should be interpreted as the result of combined interactions between microplastics and processes occurring in the rhizosphere. The analysis focused on the microbial response and did not include a direct assessment of polymer degradation processes. Despite these limitations, the applied model enabled a comparative evaluation of bioaugmentation and biostimulation, as well as an analysis of the microbiome under exposure to microplastics with different biodegradability.

## 4. Materials and Methods

### 4.1. Materials

#### 4.1.1. Characteristics of the Sampling Site

The soil samples used in the study were collected from an agricultural area located in northeastern Poland, in the western part of the Masurian Lake District, which represents a young glacial landscape. This region shows significant lithological and geomorphological variability, which results in the dominance of soils developed from morainic and fluvioglacial formations. These soils are characterized by a diverse granulometric composition and relatively high productive potential. The soils of this region, used for agriculture through long-term cereal cultivation, are developed mainly on moraine formations. They are characterized by a clay and silt content of 20–40%, which promotes water retention and fertility. This area is influenced by a cool temperate climate. The growing season lasts an average of 190–210 days, which determines the direction of agricultural production. The analyzed soil was classified as Eutric Cambisol, one of the dominant soil types in the young glacial regions of Europe, including agriculturally used soils [97]. Soil samples were collected from the topsoil layer (0–20 cm), which is the most biologically active part of the soil profile and is therefore crucial for assessing soil condition.

#### 4.1.2. Soil Characteristics

The particle size distribution of the soil used in the experiment classified it as sandy loam, with a pH of 5.15, and was as follows: sand 0.05–2.0 mm—53.00%, silt 0.02–0.05 mm—41.43%, and clay < 0.002 mm—5.06%. From the set of chemical and physicochemical properties, in accordance with research guidelines, the following were determined: hydrolytic acidity (HAC)—19.88 mmol (+) kg^−1^ d.m. and exchangeable base cations (EBC)—68.00 mmol (+) kg^−1^ d.m. Based on HAC and EBC, two soil parameters were determined: cation exchange capacity (CEC)—88.25 mmol (+) kg^−1^ d.m. and alkaline cation saturation (ACS)—77.06%. The following instruments were used in the analyses: for granulometric composition—Malvern Mastersizer 3000 Laser Diffraction (Malvern, Worcestershire, UK), for pH—HI 2221 pH meter (Hanna Instruments, Washington, UK) (soil pH) [98]. The granulometric composition analysis was performed using the laser diffraction method, determining the particle size distribution based on light scattering. pH was determined potentiometrically in 1 mol KCl dm^−3^. To determine HAC and EBC, Kappen’s method [99] was used, based on the alkaline extraction of humic substances from the soil material and their fractionation after acidification.

#### 4.1.3. Materials for Testing

The study utilized a biodegradable mulch film made from ecovio^®^ M2351 (BASF SE, Ludwigshafen, Germany), a composite of polylactic acid (PLA) and a biodegradable poly(butylene adipate-co-terephthalate) copolyester (PBAT, ecoflex^®^ Ludwigshafen, Germany). This material is intended for agricultural applications and meets the requirements of the EN 17033:2018 [100] standard regarding biodegradability in soil. The film used was 15 µm thick.

The study also planned to use a non-biodegradable film made of low-density polyethene (LDPE). LDPE is a polymer commonly used in horticulture and agriculture for soil protection and crop mulching. Low-density polyethylene (LDPE) belongs to a group of polymeric materials used in the production of plastic products, which in the European Union are subject to the requirements set forth in Regulation (EU) No. 10/2011 concerning materials intended to come into contact with food. This regulation specifies, among other things, the permissible material components and migration limits for substances, while compliance is confirmed by Declarations of Conformity (DoC) issued by manufacturers [101,102]. The film used measured 3 m × 5 m and was 150 µm thick (PRAPO, Allegro, Poland). Before being introduced into the soil, both materials were mechanically shredded into 5 mm × 5 mm fragments. The fragmentation process yielded particles classified as secondary microplastics (<5 mm) [103]. Particle size is a key factor influencing microbial colonization and migration in soil, which may affect the observed results and their interpretation.

#### 4.1.4. Isolate *Pseudomonas umsongensis* (B)

**Figure 11:**
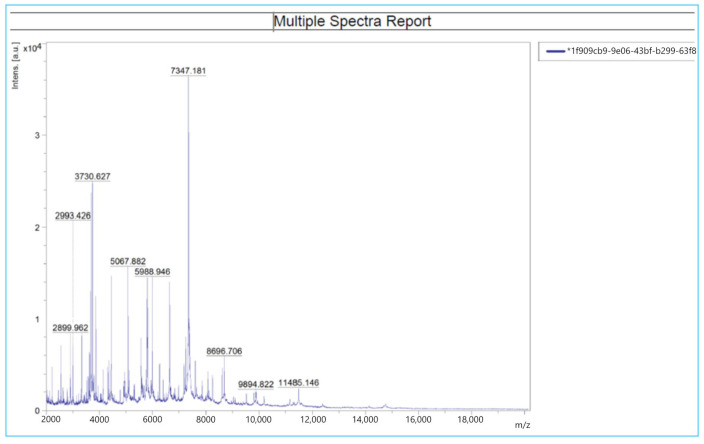
Mass spectrum of the *Pseudomonas umsongensis* strain obtained by MALDI-TOF MS. The asterisk (*) denotes a system-generated unique identifier (GUID) used to track measurement data.

*Pseudomonas umsongensis* is a strain with high metabolic versatility, capable of utilizing PET-derived monomers such as terephthalate and ethylene glycol, as confirmed in genomic and applied studies [57,104]. The isolate used in the present study was obtained from soil contaminated with bisphenol A at a level of 800 mg BP kg^−1^ d.m. of soil. The soil had a moisture content corresponding to 50% of the capillary water-holding capacity and was incubated at a constant temperature of 28 °C for 30 days. From the bacterial colonies proliferated and isolated on the medium described by Borowik et al. [105], only representative colonies were selected and purified by tenfold subculturing. Species-level identification was performed using MALDI-TOF MS (matrix-assisted laser desorption/ionization time-of-flight mass spectrometry). Identification of the bacterial isolate was performed using a Bruker MALDI Biotyper system (Bruker Daltonics, Germany) based on protein mass spectra obtained from whole-cell bacterial extracts. The obtained log(score) value of 2.25 indicated reliable species-level identification according to the manufacturer’s criteria. Contemporary studies indicate that this technique can be used not only for species-level identification but also, with expanded reference databases, for discrimination at the subspecies and strain levels [106]. The analysis involved ionization of cellular proteins using a laser pulse in the presence of an organic matrix, followed by measurement of ion time-of-flight in a time-of-flight analyzer. The obtained mass spectrum constituted a characteristic profile of the analyzed microorganism. For taxonomic identification, it was compared with a reference database (Figure 11). An isolate classified as *Pseudomonas umsongensis* and meeting the criteria for reliable species-level identification was selected for further studies. Cell proliferation of *Pseudomonas umsongensis* was conducted for 72 h in 500 cm^3^ Bunt and Rovira medium [107]. After 72 h incubation at 28 °C, all cultures were combined into a single sample, yielding a suspension containing 5.3 × 10^9^ cells cm^−3^.

#### 4.1.5. Humic Acids (H)

HumiAgra (AgraPlant, Kielce, Poland) is an organic humus-based powder obtained via hydrolytic-oxidative conversion of lignosulfonates. It contains a high proportion of humic substances (90% dry weight), including humic and fulvic acids in a 1:1 ratio, has an alkaline pH (8–10), and contains minerals such as K_2_O (8%) and S (3%). The preparation is used as a soil and plant biostimulant agent.

#### 4.1.6. Experimental Setup

The experimental procedure adopted in this study was selected to eliminate the influence of factors interfering with changes in the structure of microorganisms. Determining this parameter in soil subjected to microplastic stress was the primary research objective. Therefore, the experiment was conducted in a vegetation hall (53°45′36″ N, 20°27′15″ E), with air temperature and soil moisture (maintained at 60%) monitored throughout the 60-day experimental period. In the first step, the soil was sieved through a 0.5 cm mesh sieve and then packed, together with the added contaminants and biostimulatory substances, at a rate of 3.4 kg per pot. The experimental factors included: type of secondary microplastic—biodegradable mulch film (PLA) and non-biodegradable low-density polyethylene film (LDPE) (1); dose of PLA and LDPE applied at two levels: 0 g (control) and 4 g (2); and type of biostimulation: bioaugmentation with *Pseudomonas umsongensis* (B) and biostimulation with humic acid preparation HumiAgra (H). The selection of this contamination level was based on the results reported by Zhang and Liu [108], which indicated high spatial heterogeneity of microplastics, with reported values ranging from 7100 to 42,960 microplastic particles kg^−1^ d.m. of soil, confirming a tendency for the formation of local zones of elevated accumulation. The applied level of contamination reflects conditions of strong environmental stress in experimental conditions. Humic acids were applied at a dose of 2 g kg^−1^ dry soil weight. The *Pseudomonas umsongensis* inoculum was applied as 10 cm^3^ of liquid culture suspension, corresponding to 5.3 × 10^10^ kg^−1^ of soil. In control treatments, the same volume of sterile medium used for isolate proliferation was introduced to exclude its effect on soil microbial activity. The use of commercially available mulch films aimed to reflect real environmental conditions associated with the application of polymers in agricultural practice. Therefore, the soil was sown with maize (*Zea mays*) of the DS1897B cultivar. Consequently, all experimental treatments received macronutrient fertilization at rates corresponding to optimal plant nutritional requirements: N—150, P—150, K—50, and Mg—20 mg·kg^−1^ soil. Throughout the 60-day experiment, a density of four plants per pot was maintained, and harvesting was performed at BBCH stage 59 (Biologische Bundesanstalt, Bundessortenamt und Chemical industry). *Zea mays* was included in all experimental treatments to simulate agricultural conditions; therefore, plant-related effects were not treated as a separate experimental factor. The obtained fresh biomass yield of *Zea mays* is presented in Table 1.

#### 4.1.7. Cultivable Bacteria

In this experiment, the abundance (cfu), colony development indices (CD) [109], and ecophysiological diversity indices (EP) [110] of organotrophic bacteria (Org) and actinomycetes (Act) were determined. The selection of organotrophic bacteria is justified by their high proliferative capacity. They also play a key role in organic matter decomposition, highlighting their fundamental function in humification and mineralization processes, as well as their responsiveness to anthropogenic contaminants, including microplastics [65]. In contrast, actinomycetes exhibit potential for microplastic degradation through enzymatic activity and biofilm-mediated colonization of microplastic surfaces [110].

The analysis was performed using the pour plate method with serial dilutions for Org and Act (10^−4^ and 10^−5^), resulting in four replicates for each sample. Proliferation was carried out on Bunt and Rovira media [106] for Org and on Küster and Williams medium [111] for Act. To determine CD and EP indices, colonies of Org and Act were counted over a 10-day incubation period at a constant temperature of 28 °C (PSelecta Incudigit Incubator, Barcelona, Spain).

#### 4.1.8. Isolation of Bacterial DNA and Identification of Non-Cultivable Bacteria

In subsequent stages, genomic DNA was extracted using the MagnifiQ™ 1 Genomic DNA Instant Kit (A&A Biotechnology, Gdynia, Poland) (1), a FastPrep-24 instrument for mechanical homogenization enabling cell lysis (30 s, 6 m/s) (2), and the Anti-Inhibitor Kit (A&A Biotechnology, Gdańsk, Poland), which was used for the removal of substances inhibiting PCR and real-time PCR reactions (3). DNA concentration was quantified using a Qubit 4 Fluorometer (Thermo Fisher Scientific, Waltham, MA, USA) (4). The presence of DNA was confirmed by real-time PCR. Amplification detection was performed using SYBR Green dye, which emits a fluorescent signal during the PCR reaction. Bacterial identification was performed by amplifying the V3–V4 region of the 16S rRNA gene using next-generation sequencing (NGS) technology. For this purpose, Illumina adapter sequences (5′ → 3′) were used: 341F: TCGTCGGCAGCGTCAGATGTGTATAAGAGACAG and 785R: GTCTCGTGGGCTCGGAGATGTGTATAAGAGACAG. Following sequencing on the Illumina MiSeq platform, approximately 50,000 paired-end reads per sample were obtained and subjected to quality filtering. The resulting sequences were taxonomically assigned at the species level using the QIIME 2 version 2023.5 [112] software package and the Greengenes reference database. Based on the obtained data, biodiversity indices were calculated, including the Shannon–Wiener index (H), Simpson’s index (S), and Margalef’s richness index (Dm).

### 4.2. Statistical Analysis and Data Processing

To identify homogeneous groups and compare mean values, Tukey’s HSD test was applied at a significance level of *p* = 0.05, accounting for unequal sample sizes (N) [113]. Alpha diversity indices at the genus level were calculated according to the methodologies described by Jiang et al. [114] for Margalef’s richness index (D_m_), and by Yang et al. [115] for the Shannon–Wiener index (H’) and Simpson’s index (S). Bacterial metabolic and ecological functions were assigned based on taxonomic profiles using the FAPROTAX database [116], implemented within the MACADAM platform [117]. These predictions reflect potential functions derived from taxonomic composition and do not represent directly measured activity. Relationships between representative genera in soil and bacterial metabolic and ecological functions were assessed using Spearman’s rank correlation coefficient [113]. All experimental data were processed using the following software: Statistica 13.3 [113], TBtools-II Version: 2.4 [118], R software v1.2.5033 (Boston, MA, USA) with R v3.6.2 [119], and the online data visualization platform SRplot (bioinformatics.com.cn) [119].

## 5. Conclusions

The conducted research expands understanding of the extent of disturbances caused by PLA and LDPE mulch films in soil. The results indicate that microplastics derived from PLA and LDPE significantly alter the abundance, structure, and function of the soil microbiome in agriculturally used soil. The response of cultured bacteria revealed two significant trends: high sensitivity of organotrophic bacteria to both microplastics, with PLA exhibiting a stronger inhibitory effect, and a positive response of actinomycetes to the presence of both PLA and LDPE.

The degree of biodegradability of the mulching materials was a significant factor shaping the structure of the soil microbiome. The results indicate that PLA and LDPE differentiated the responses of the main taxonomic groups, while maintaining the dominance of *Proteobacteria*, *Actinobacteriota*, *Acidobacteriota*, and *Chloroflexi* across all experimental variants. PLA primarily promoted an increase in the proportion of *Actinobacteriota*, whereas in the case of LDPE, an increase in the proportion of *Actinobacteriota* and *Chloroflexi* was observed, accompanied by a decrease in diversity expressed by the number of ASVs. Changes also affected lower taxa: in the PLA variant, *Bacillus* and *Limnochorda* were more common, whereas in LDPE-treated soil, the proportion of *KD4-96* and *1921-2* increased, indicating the selective nature of the studied polymers’ effects.

Both microplastics also provided a microbial niche conducive to the presence of taxa of potential pathogenic or opportunistic significance, including bacteria of the genera *Bacillus*, *Mycobacterium*, *Ralstonia*, and *Cupriavidus*. In the PLA treatment, unlike in the LDPE treatment, an additional increase was observed in the abundance of taxa such as *Leifsonia* sp. and *Curtobacterium* sp., which are described in the literature as potentially associated with phytopathogenicity. Both PLA and LDPE significantly reduced the abundance of *Enterobacter* sp. and *Herbaspirillum* sp.

In response to the observed changes in the soil microbiome, two approaches were applied to mitigate the effects of microplastic exposure. Bioaugmentation using the *Pseudomonas umsongensis* strain was an innovative approach and demonstrated higher efficacy than biostimulation with humic acids. The results indicate that the effectiveness of bioremediation strategies depends on their nature, reflecting differences in their mechanisms of action. It is worth noting that the obtained results reflect the short-term response of the soil microbiome to the presence of mulching materials in the soil, which is consistent with the seasonal nature of their application; however, due to the possibility of accumulation of their fragments in the soil environment, further research on the persistence of the observed changes is warranted.

## Figures and Tables

**Table 1:** Aboveground biomass yield of *Zea mays*, g f. m. pot^−1^ in soil contaminated with PLA and LDPE.

Treatment	Type of Biostimulation of Soil			Average
	Control	*Pseudomonas* *umsongensis*	Humic Acids	
C	378.75 ± 0.58 ^i^	382.50 ± 0.96 ^h^	447.50 ± 1.29 ^d^	402.92 ^B^
PLA	407.50 ± 0.58 ^g^	421.25 ± 1.50 ^f^	470.00 ± 1.41 ^b^	432.92 ^AB^
LDPE	435.00 ± 0.82 ^e^	458.75 ± 1.50 ^c^	526.25 ± 0.96 ^a^	473.33 ^A^
Average	407.42 ^Y^	420.83 ^XY^	481.27 ^X^	

C—uncontaminated soil; PLA—soil contaminated with biodegradable mulch film; LDPE—soil contaminated with low-density polyethylene mulch film. Homogeneous groups denoted with letters (a–i) were calculated for soil biostimulation and microplastic treatments. Uppercase letters A and B indicate significant differences in mean values for microplastics, whereas letters X and Y indicate significant differences in mean values for biostimulant substances.

## Data Availability

The original contributions presented in this study are included in the article. Further inquiries can be directed to the corresponding author.

## Abbreviations

The following abbreviations are used in this manuscript:

PLA	Biodegradable PLA mulch film
LDPE	Low-density polyethylene (LDPE) film
C	Uncontaminated soil
B	Soil bioaugmented with *Pseudomonas umsongensis*
H	Soil biostimulated with humic acids
S	Simpson index
Dm	Margalef richness index
H’	Shannon–Wiener index
